# Tranexamic Acid for Adults with Melasma: A Systematic Review and Meta-Analysis

**DOI:** 10.1155/2018/1683414

**Published:** 2018-11-06

**Authors:** Lei Zhang, Wei-Qiang Tan, Qing-Qing Fang, Wan-Yi Zhao, Qi-Ming Zhao, Jie Gao, Xiao-Wei Wang

**Affiliations:** ^1^Department of Orthopaedics, Xiaoshan Hospital of Traditional Chinese Medicine, Hangzhou 311201, China; ^2^Department of Plastic Surgery, Affiliated Sir Run Run Shaw Hospital to Zhejiang University School of Medicine, Hangzhou 310016, China; ^3^Department of Plastic Surgery, The First Affiliated Hospital to Zhejiang University School of Medicine, Hangzhou 310003, China; ^4^Department of Plastic Surgery, Zhejiang Hospital, Hangzhou 310013, China

## Abstract

**Objective:**

Melasma is a highly prevalent, chronic, and pigmentary disorder. This systematic review aims to evaluate the efficacy and safety of tranexamic acid (TA) for the treatment of adults with melasma.

**Methods:**

We independently searched 3 databases from beginning to 26 April, 2018. The study included 21 eligible trials. Two writers extracted data at the same time independently. Study outcomes were calculated by standardized mean differences (SMD) with 95% confidence intervals (CIs). All statistical analyses were performed using Review Manager Version 5.3 and STATA Version 15.1.

**Results:**

The combined results showed that the use of TA was associated with reduced Melasma Area and Severity Index (MASI) and Melanin Index (MI). No significant difference in Erythema Index (EI) was observed with TA treatment. Side effects were minor, with a few cases reporting mild gastrointestinal reaction, oligomenorrhoea, hypopigmentation, urticarial rash, and skin irritation xerosis.

**Conclusion:**

The meta-analysis suggested that TA treatment appeared to be a promising therapeutic approach for melasma.

## 1. Introduction

Melasma, also referred to as chloasma, is a common acquired condition of pigmentary disorder marked by irregular hyperpigmented macules or patches and most commonly occurs in females with dark skin types living in areas of intense ultraviolet (UV) light exposure. The prevalence of melasma reported in recent studies ranges from 8.8% to 40% based on ethnic makeup of the population [1, 2]. Many factors are linked with the development of melasma, including UV radiation, pregnancy, hormonal activity, thyroid abnormalities, and medications [3]. Furthermore, there appears to be a genetic predisposition of melasma. All these diverse factors trigger the increased synthesis of melanosomes in melanocytes and increased transfer of melanosomes to keratinocytes [4].

The treatments for melasma are generally aimed at inhibiting the pathways that synthesize melanin and decrease of melanosome transfer from melanocyte to keratinocytes. Because both UV and visible light can induce pigmentation, the therapy usually starts with the protection of UV sun, and topical lightening formulation. Moreover, various subsequent treatments of melasma include hypopigmenting agents, chemical peels, lasers, and dermabrasion [5]. However, melasma is often difficult to treat and can be psychosocially detrimental to many patients [6–9]. Some topical agents, such as hydroquinone, are limited by complications including irritant dermatitis, allergic contact dermatitis, postinflammatory hyperpigmentation, nail bleaching, and exogenous ochronosis [2]. Furthermore, physical treatments such as chemical peels and low-fluence Q-switched neodymium-doped yttrium aluminum garnet laser (QSNY) need multiple courses over several months. However, the side effects and financial burden of these treatments limit their clinical application [10].

Tranexamic acid (trans-4-(Aminomethyl)cyclohexanecarboxylic acid, TA), a plasmin inhibitor, is used as a hemostatic agent to treat abnormal fibrinolysis to prevent excessive bleeding. It is a synthetic derivative of the amino acid lysine and exerts its effect by competitively inhibiting the activation of plasminogen activator (PA) through reversible interactions with its lysine-binding sites, thus inhibiting PA from converting plasminogen to plasmin [32]. TA is a relatively new drug for melasma and was first reported in 1979 when Nijo Sadako tried to use it to treat a patient with chronic urticaria. This was an accidental finding but prompted studies of TA on melasma patients [33]. As a skin-lightening agent, TA has been used as a topical, intradermal microinjection, and oral agent [11]. Although TA has emerged as a potential treatment for melasma, it has not been approved by Food and Drug Administration of the United States for melasma and treatment remains controversial [10, 33, 34]. Consequently, in this study, we conducted a systematic review to evaluate the therapeutic effect of TA for treating melasma.

## 2. Materials and Methods

The protocol of this systematic review and meta-analysis was registered in advance with the PROSPERO international prospective register of systematic reviews (registration number CRD42018095168) and conforms to the Preferred Reporting Items for Systematic Review and Meta-analysis (PRISMA) statement [35]. This study does not require an ethical review.

### 2.1. Database and Search Strategy

In this study, the PubMed (https://www.ncbi.nlm.nih.gov/pubmed), EMBASE (https://www.embase.com/), and the Cochrane Library (https://www.cochranelibrary.com/) databases were searched for original studies and the search was conducted on 26 April 2018. The following keywords and their combinations were used: “melasma”, “chloasma”, “tranexamic acid”, “antifibrinolytic agents”, and “trans-4-(Aminomethyl)cyclohexanecarboxylic acid”.

### 2.2. Inclusion and Exclusion Criteria

Two reviewers (LZ and XWW) independently qualified all studies, and if the evaluations did not yield a final decision, a third reviewer (WQT) was invited to make a decision. Qualified studies had to satisfy the following inclusion criteria: (1) original studies (randomized controlled trials (RCTs), cohort studies, and case-control studies) describing treatment for melasma with TA; (2) TA treatment either alone or in combination with other treatments; (3) studies reporting one of the melasma outcome measures such as the Melasma Area and Severity Index (MASI), the Melanin Index (MI), and Erythema Index (EI). Principal criteria for the exclusion of studies were as follows: (1) irrelevant topic, duplicate, review, and comment; (2) no appropriate or complete data; (3) studies not reporting melasma specific outcome measures.

### 2.3. Data Extraction

All data from the qualified studies were independently extracted by two reviewers (LZ and XWW). The following types of data were extracted: study characteristics (first author, publication year, countries/regions, and sample size of participants), pharmacotherapy intervention, administration methods, duration of follow-up, outcomes (MASI, MI, and EI), and adverse reaction. Any disagreement was resolved by discussion.

### 2.4. Quality Assessment

RCTs were evaluated by the Cochrane risk-of-bias tool [36]. And we adopted the risk-of-bias criteria suggested by Newcastle-Ottawa Scale for case-control studies and cohort studies (http://www.ohri.ca/). Two reviewers (LZ and XWW) independently assessed methodological quality of each study. Any disagreements were resolved by consensus or by consultation with a third review (WQT).

### 2.5. Statistical Analysis

The Review Manager Version 5.3 (the Nordic Cochrane Centre, the Cochrane Collaboration, Copenhagen, Denmark) and STATA Version 15.1 software (StataCorp, TX, USA) were used for data analysis. Our main indicators of treatment for melasma by TA were the difference in MASI, MI, and EI scores before and after the treatment and the difference in MASI score between a routine treatment and that with TA as an adjuvant. We analyzed the data using a random-effect model, and heterogeneity across pooled studies was tested using Cochrane Q via a Chi^2^ test, quantifying with the *I*^2^ statistic. And *P* < 0.5 and *I*^2^ > 50% indicate a significant heterogeneity between studies. The continuous outcome data were expressed as standardized mean differences (SMD) and the 95% confidence intervals (CIs) were also calculated. Subgroup analyses were conducted in these studies by the TA administration (oral TA, topical TA, and TA injection). Egger test was carried out to investigate the publication bias of enrolled studies.

## 3. Result

### 3.1. Search Results

**Figure 1:**
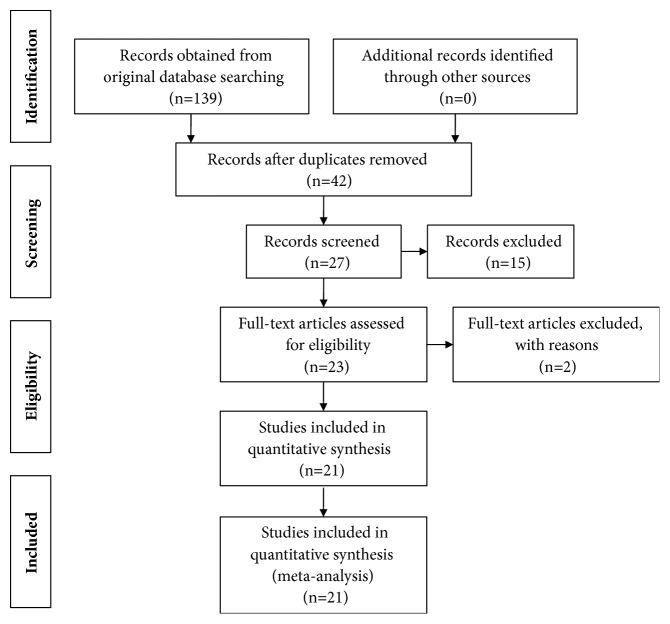
Flow diagram of the meta-analysis.

We used a flow chart to make the search process more detailed (Figure 1). A total of 139 articles in English were retrieved for this study from databases specified in Section 2.1. About 97 articles were excluded for duplication. After reviewing the title and abstract, 15 articles were excluded for reviews. Finally, the remaining 23 full-text articles were assessed for eligibility. Additional 2 articles were excluded for the lack of important outcomes. Eventually, 21 studies were incorporated into the information integration [11–30, 37].

### 3.2. Study Characteristics

The main characteristics of these included studies are shown in Table 1. The included studies were published between 2006 and 2018, which were comprised of 16 RCTs, 3 cohort studies, and 2 case-control studies. Among these 21 trials, 6 were conducted in Korea, 5 in Iran, 4 in India, 2 in China, 1 in Brazil, 1 in Nepal, 1 in Singapore, and 1 in USA. These studies involved a total of 1563 patients with melasma. All patients in these studies were adults, 80%-100% women. Melasma severity was evaluated by MASI, MI, and EI (12 studies were assessed with the original MASI score, 6 studies were assessed with the modified MASI score, 3 studies were assessed with the MI score, and 3 studies were assessed with EI score). TA was delivered orally, topically, and through physical methods in these articles. The oral groups patients received a daily dose of 500mg or 750mg [11, 13, 15, 18–22, 24, 27]. And the dose of injection TA ranged from 2-8mg to the entire affected area or was 0.2mg/cm^2^ in affected area [12, 17, 24, 27–29]. In addition, the form of topical TA included liposome [14], emulsion/cream [16, 29], skin lotion [16, 25, 37], and cataplasm [30]. And 0.5%-5% TA was administrated to each patient in the topical group [14–16, 23, 25, 26, 29, 30, 37]. The mean treatment time was approximately 8 to 12 weeks.

### 3.3. Quality Assessment

The quality of the studies is evaluated respectively by the Cochrane risk-of-bias tool in Table 2 and Newcastle-Ottawa Scale in Tables 3 and 4. The quality of the studies evaluating TA treatment in melasma varied. However, only a few studies were assessed as having high quality.

### 3.4. Effects of Interventions

#### 3.4.1. Comparison of MASI Score Change

**Figure 2:**
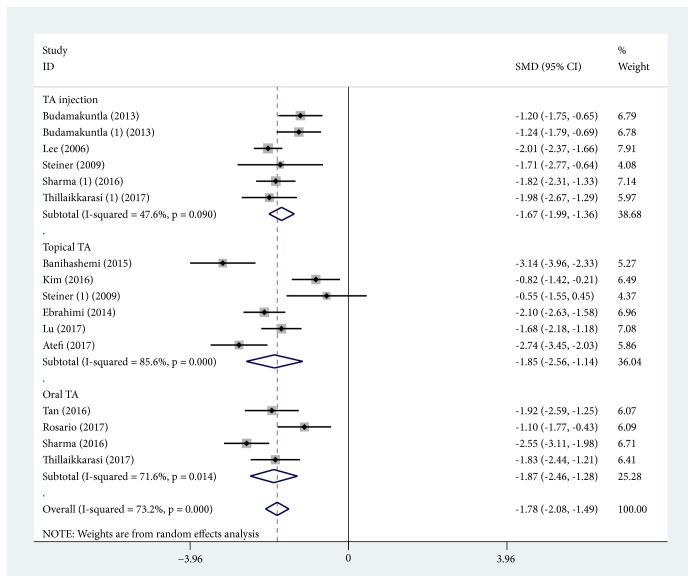
Forest plot showing the comparisons of Melasma Area and Severity Index (MASI) score change between post- and pre-tranexamic acid (TA) treatment alone.

**Figure 3:**
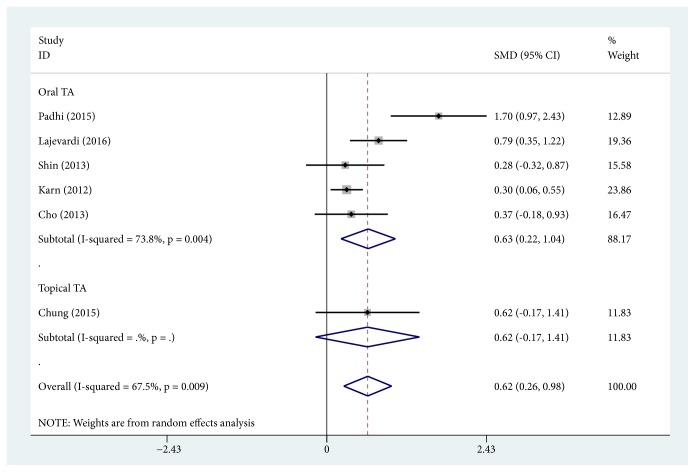
Forest plot showing the comparisons of Melasma Area and Severity Index (MASI) score change between the routine treatment combined with or without tranexamic acid (TA) adjuvant treatment.

MASI score reduction, defined as change in MASI score before and after treatment, was the primary outcome measure of 12 studies with TA treatment alone [12, 14, 16, 17, 20, 21, 23–25, 27, 29, 30]. Among these studies, 5 trials [12, 17, 24, 27, 29] with TA injection were pooled together using the random effects model (SMD, -1.673; 95% CI, -1.990 to -1.356; *P* < 0.0001; *I*^2^ = 47.6%). Six trials with topical TA were pooled together to be analyzed [14, 16, 23, 25, 29, 30], and the random effects model was applied (SMD, -1.850; 95% CI, -2.556 to -1.144; *P* < 0.0001; *I*^2^ = 85.6%). Furthermore, 4 pooled trials [20, 21, 24, 27] with oral TA were used by the random effects model (SMD, -1.866; 95% CI, -2.456 to -1.276; *P* < 0.0001; *I*^2^ = 71.6%). Overall, TA treatment alone induced a significant reduction of MASI score (SMD, -1.783; 95% CI, -2.076 to -1.490; *P* < 0.0001; *I*^2^ = 73.2%). There was high and statistically significant heterogeneity within TA treatment alone subgroups, as shown in the wide confidence intervals (Figure 2). However, there was no heterogeneity among 3 subgroups (*I*^2^ = 0%). MASI score change was also compared to the routine treatment of melasma combined with or without TA adjuvant treatment in 6 studies [11, 13, 18, 19, 22, 26]. Among these studies, routine treatment included low-fluence 1064-nm quality-switched neodymium-doped yttrium aluminum garnet (QSNY) [11, 22], topical 4% hydroquinone [13, 18, 19], and intense pulsed light (IPL) [22, 26]. Oral TA was applied as an adjuvant treatment in 5 studies [11, 13, 18, 19, 22]. And topical TA was applied only in 1 study [26]. The results of the 5 pooled trials with oral TA adjuvant treatment using the random effects model (SMD, 0.629; 95% CI, 0.216 to 1.042; *P* = 0.003) showed high heterogeneity (*I*^2^ = 73.8%), suggesting that MASI score reduction was greater in the subgroup of TA adjuvant treatment. Additionally, topical TA adjuvant treatment was also found to reduce the MASI score (SMD, 0.618; 95% CI, -0.171 to 1.406), but the difference was not statistically significant (*P* = 0.125). Taken altogether, the heterogeneity was high within the TA adjuvant treatment subgroup (*I*^2^ = 67.5%) (Figure 3), but there was no heterogeneity between the 2 subgroups (*I*^2^ = 0%).

#### 3.4.2. Comparison of MI Score Change

**Figure 4:**
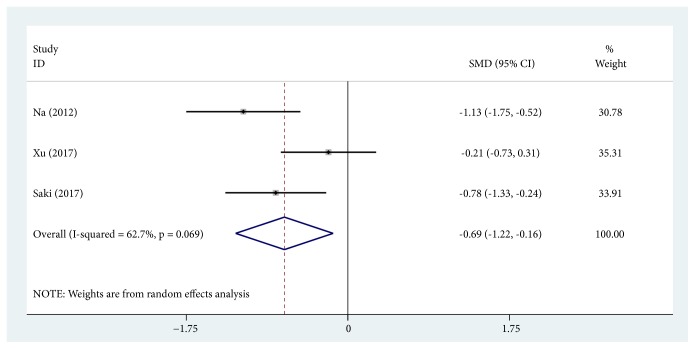
Forest plot showing the comparisons of Melanin Index (MI) score change between post- and pre-tranexamic acid (TA) treatment alone.

MI score change was defined as change in MI score before and after treatment. Three trials were pooled together [15, 28, 37] using the random effects model (SMD, -0.689; 95% CI, -1.217 to -0.160; *P* = 0.011; *I*^2^ = 62.7%), suggesting that TA treatment significantly decreased MI score (Figure 4).

#### 3.4.3. Comparison of EI Score Change

**Figure 5:**
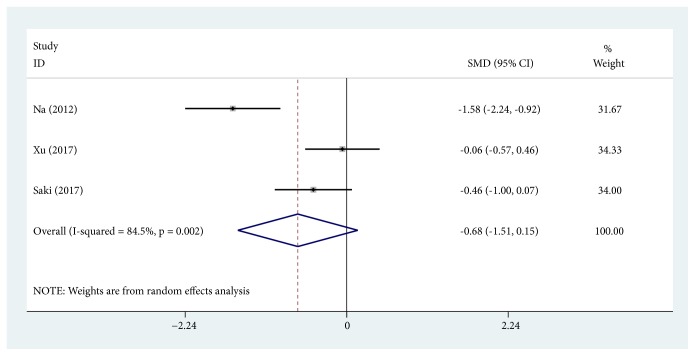
Forest plot showing the comparisons of Erythema Index (EI) score change between post- and pre-tranexamic acid (TA) treatment alone.

Similarly, EI score change was also defined as change in EI score before and after treatment. Three trials were pooled together [15, 28, 37] using the random effects model (SMD, -0.677; 95% CI, -1.507 to 0.153; *P* = 0.110; *I*^2^ = 84.5%). There was no significant difference in EI score measured before and after TA treatment (Figure 5).

### 3.5. Side Effects

In the included 21 studies, 10 trials were related to the side effects (Table 1). The side effects of patients with melasma after oral TA application mainly showed gastrointestinal reaction such as heartburn, nausea, abdominal pain, and epigastric discomfort [11, 13, 19, 24]. And some patients showed oligomenorrhoea, hypopigmentation, urticarial rash with angioedema, moderate myalgias, transient headache, anxiety, and depression [18, 21, 22]. Additionally, patients with topical TA treatment showed side effects such as skin irritation xerosis, and scaling [25]. The patients with TA injection just showed transient oedema and injection site pain [24]. However, small number of erythema cases appeared in all oral, topical, and injectional TA subgroups [12, 18, 25].

### 3.6. Publication Bias

**Figure 6:**
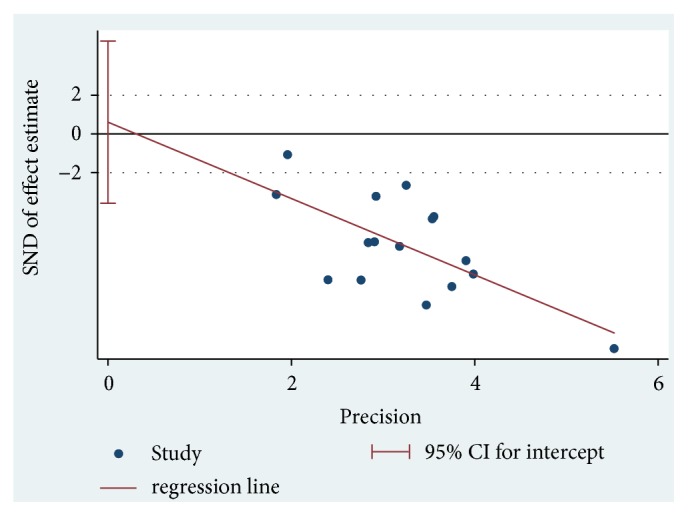
The publication bias of tranexamic acid (TA) treatment with Egger funnel plot.

The included articles' publication bias was evaluated through STATA 15.1 software. Because both of the indicators of MI and EI only had 3 articles, they did not meet the standard of the funnel map, which was without special significance. And we assessed the publication bias to the MASI score by using the Egger method. The results showed that the MASI score comparison showed no significant publication bias (*P* = 0.760) (Figure 6, Table 5).

## 4. Discussion

This meta-analysis of individual patient data from 1563 adults regardless of sex randomized in trials of single and adjuvant TA found a highly significant reduction in MASI and MI scores, but not in EI score. Subgroup analysis suggested benefit in oral, topical, and injectional TA alone and adjuvant oral TA; among whom there were highly significant reductions in MASI score. However, adjuvant topical TA did not show a significant difference in MASI score compared with the routine treatment because just one study was included [26].

TA was often applied to inhibit bleeding by its effects on blocking plasminogen activation and thus subsequently stopping fibrinolysis [31, 38]. Although the indications for TA did not include treatment of patients with melasma, the potential efficacy for melasma has been consistently reported since the 1980s. It is generally known that melasma is an acquired disorder of symmetrical hyperpigmentation. UV radiation induces the synthesis of PA by keratinocytes, which results in increased conversion of plasminogen to plasmin [39]. TA can suppress UV induced epidermal melanocyte tyrosinase activity by blocking the interaction of melanocytes and keratinocytes through the inhibition of the plasminogen/plasmin system [32]. However, TA treatment for melasma remains controversial.

A recent meta-analysis published during the last few years assessed the effectiveness and safety of TA for melasma [40]. Compared with previous systematic review, 12 studies [11, 13–16, 21, 23, 24, 27, 28, 30, 37] of this review were newly included. And most of these articles are published in 2017 or 2018. Importantly, we conducted a subgroup analysis in different administrations of TA and publication bias analysis which was performed by Egger test. Moreover, besides MASI score, more indicators (MI and EI) for melasma were also detected in this systematic review. The MASI, an index devised to accurately quantify the severity of melasma and changes during therapy, was first used by Kimbrough-Green et al. [41]. Experts in melasma have used the MASI score as the predominant outcome measure for almost 20 years, suggesting that it has content validity as well [6, 8, 41, 42]. Although individual components are problematic, particularly assessment of homogeneity and chin, MASI score system is still the most commonly used outcome measure for melasma [43]. MI score is regarded as a parameter which is mainly influenced by the melanin content [44]. Compared with whole-face assessment of MASI score, MI and EI scores are measured in melasma area (lesional skin area) by reflectance instruments [15, 28, 31]. Besides, some trials included in this study did not use the MASI score because it is composed of scores of areas and severity of the whole face, so it cannot be used for split-face assessments [31].

In our study, we proved that TA is a safe and efficient medicine for melasma patients. Both single and adjuvant TA treatments showed a significant reduction of MASI and MI scores while incurring minimal side effects. However, the combined results of 3 trials [15, 28, 37] showed no significant difference in EI score with or without TA treatment. Because of design differentials, it cannot be confirmed that TA cannot induce EI reduction in melasma patients. It is known that plasmin plays an important role in angiogenesis, as it converts extracellular matrix-bound vascular endothelial growth factor into freely diffusible forms, whereas TA inhibits angiogenesis and also suppresses neovascularization induced by basic fibroblast growth factor [37]. More high-quality trials will be needed to provide strong evidence of the effect of TA for EI reduction.

The daily systemic doses of TA reported for the treatment of melasma have ranged from 500mg to 700mg, and the effective daily dose is much lower for menorrhagia and perioperative hemophilia patients [32, 39]. Nevertheless, given the potential for serious side effects with the use of oral TA, there has been interest in evaluating injection or topical TA for melasma [12, 14, 16, 17, 23, 25, 26, 28–30, 37]. Topical TA has been applied alone and in conjunction with intradermal injection, microneedling, fractionated CO_2_ laser, and so on to increase bioavailability [28, 37, 45]. However, the currently available data are limited by small sample sizes and the design of poor quality. Debate exists about whether topical water-soluble TA is transdermally absorbed by the lipid-soluble surface of the skin. In addition to bridging the knowledge gaps in our understanding of topical as an option for melasma patients, further studies on clinical efficacy and side effects comparisons among different administrations of TA should be carried out.

There are some limitations of this meta-analysis and system review which are as follows. Firstly, trials included in this meta-analysis had various follow-up times and different doses and used different forms of MASI score. Secondly, the quality of studies was low and assessment of the MASI score may be influenced by the subjective factors in the process. In addition, there was greater heterogeneity of MASI, MI, and EI in this systematic review which may be due to sample size and quality of studies. However, it is worth noting that high heterogeneity is more common in continuous variables [46]. We conducted a subgroup analysis in different TA modes of administration, and different degrees of reductions of intra-subgroup *I*^2^ were observed. However, it is difficult to evaluate the heterogeneity in terms of individual differences. Despite the disadvantages mentioned above, this systematic review and meta-analysis provides preliminary evidence currently available on the application of TA for melasma patients.

## 5. Conclusion

According to the results of meta-analysis, TA is an effective and safe therapy for melasma patients. However, the findings are insufficient to make a firm conclusion due to the lack of studies with high methodological quality. More rigorously designed trials with larger sample size are needed to confirm the effectiveness and safety of TA for melasma.

## Figures and Tables

**Table 1:** The characteristics of included studies.

Study (first author, year)	Country	Completed/Participants	Age, y	Delivery mode	TA treatment*∗*	Primary outcome^§^	Side effects (patients)
Shin 2013[11]	Korea	44/48	48^†^(28-56)	Oral	750mg (Qd, 8W) + QSNY (2 rounds, 4W intervals)	mMASI	Heartburn (1); Nausea (1)

Budamakuntla 2013[12]	India	52/60	- (18-50)	Injection	MI: 8mg (Qm, 8W) MN: 2-4mg (Qm, 8W)	mMASI	Itching (4); Burning (3); Erythema (8)

Lajevardi 2016[13]	Iran	88/100	- (18-65)	Oral	250mg (Tid, 12W) + Topical 4% HQ (Qn, 12W)	MASI	Therapy-related complications (3); Abdominal pain (2)

Banihashemi 2015[14]	Iran	23/30	- (25-47)	Topical	5% TA (Qn, 12W)	MASI	None

Na 2012[15]	Korea	22/25	- (20-55)	Oral+ Topical	Oral TA 250mg (Tid, 8W) + Topical 2% TA (Bid, 8W)	MI; EI	None

Kim 2016[16]	Korea	23/23	- (34-60)	Topical	Emulsion 2% TA (Bid, 12W) + Fabric mask 2% TA (Tiw, 12W)	mMASI	None

Lee 2006[17]	Korea	85/100	38^‡^ (29-46)	Injection	0.2mg/cm^2^ (Qw, 12W)	MASI	None

Padhi 2015[18]	India	40/40	36^‡^ (24-55)	Oral	250mg (Bid, 8W) + Topical 4% HQ (Qd, 8W)	MSAI	Erythema (2); Burning (2); Hypopigmentation/Depigmentation (2); Oligomenorrhoea (1)

Karn 2012[19]	Nepal	260/260	30^‡^ (17-55)	Oral	250mg (Bid, 12W) + Topical HQ	MASI	Oligomenorrhoea (19); Belching (12); Abdominal cramps (9); Palpitation (1); Urticarial rash with angioedema (1)

Tan 2016[20]	Singapore	25/25	47^‡^ (32-63)	Oral	250mg (Bid, 12W)	MASI	None

Rosario 2017[21]	USA	39/44	44^‡^ (-)	Oral	250mg (Bid, 12W)	mMASI	Moderate myalgias (1)

Cho 2013[22]	Korea	51/51	41^‡^ (-)	Oral	500mg (Qd, 8W) + IPL + QSNY (3-4 rounds, 1-2W intervals)	mMASI	Transient headache (4)

Atefi 2017[23]	Iran	60/60	39^‡^ (-)	Topical	5% TA (Bid, 12W)	MASI	None

Sharma 2016[24]	India	80/100	37^‡^ (18-55)	Oral Injection	Oral TA 250mg (Bid, 12W) TA Injection 4mg (3 rounds, 4W intervals)	MASI	Oral: Hypomenorrhea (6); Epigastric discomfort (2) Injection: Injection site pain and transient oedema (13)

Ebrahimi 2014[25]	Iran	39/50	40^‡^ (29-51)	Topical	3% TA (Bid, 12W)	MASI	Erythema, skin irritation, xerosis, and scaling (Total: 9)

Chung 2015[26]	Korea	13/15	41^‡^ (-)	Topical	2% TA (12W) + IPL (4 rounds, 4W intervals)	mMASI	None

Thillaikkarasi 2017[27]	India	48/60	40^‡^ (-)	Oral Injection	Oral TA 250mg (Bid, 12W) TA Injection 4mg (3 rounds, 4W intervals)	MASI	Hypothyroidism (5); Irregular menstrual cycle (7 females); Depression and anxiety (38)

Saki 2017[28]	Iran	31/37	36^‡^ (25-49)	Injection	3 rounds, 4W intervals	MI; EI	Unknown

Steiner 2009[29]	Brazil	17/18	41^‡^ (23-52)	Topical Injection	Topical 3% TA (Bid, 12W) TA Injection 0.2mg/cm^2^ (Qw, 12W)	MASI	Side effects were minimal.

Lu 2017[30]	China	81/84	43^†^(23-58)	Topical	2.5% TA (7h/d, 8W)	MASI	None

Xu 2017[31]	China	28/30	39^‡^ (20-50)	Topical	0.5% TA (Qw, 12W)	MI; EI	None

^†^Median age. ^‡^Mean age.

*∗*TA: tranexamic acid, HQ: hydroquinone, Qd: once a day, Qn: once a night, Bid: twice a day, Tid: thrice a day, Qm: once a month, Qw: once a week, Tiw: thrice a week, QSNY: low-fluence 1064-nm quality-switched neodymium-doped yttrium aluminum garnet, MI: microinjection, MN: microneedling, IPL: intense pulsed light.

^§^MASI: Melasma Area and Severity Index, mMASI: modified Melasma Area and Severity Index, MI: Melanin Index, EI: Erythema Index.

**Table 2:** Risk-of-bias assessment of included randomized controlled trials*∗*.

Study (first author, year)	Adequate sequence generation	Allocation concealment	Blinding of participants	Blinding of assessment	Incomplete outcomes data addressed	Selective reporting	Free of other bias
Atefi 2017	Low	Low	Low	Unclear	Low	Unclear	Unclear

Banihashemi 2015	Unclear	Unclear	High	Unclear	High	Unclear	Unclear

Budamakuntla 2013	Unclear	Unclear	Low	High	High	Unclear	Unclear

Chung 2015	Unclear	Unclear	High	High	Low	Unclear	Unclear

Ebrahimi 2014	Unclear	Unclear	Low	Unclear	Low	Unclear	Unclear

Karn 2012	Low	Unclear	High	High	Low	Unclear	Unclear

Lajevardi 2016	Low	Low	Low	Low	High	Unclear	Unclear

Lu 2017	Low	Unclear	Low	Low	Low	Unclear	Unclear

Padhi 2015	Unclear	High	High	Unclear	Unclear	Unclear	Unclear

Rosario 2017	Low	Low	Low	Low	Unclear	Unclear	Unclear

Saki 2017	Unclear	Unclear	High	Unclear	High	Unclear	Unclear

Sharma 2016	High	Unclear	High	Unclear	Low	Unclear	Unclear

Shin 2013	Unclear	Unclear	High	Low	Low	Unclear	Unclear

Steiner 2009	Unclear	Unclear	High	Low	Unclear	Unclear	Unclear

Thillaikkarasi 2017	Low	High	High	Unclear	Unclear	Unclear	Unclear

Xu 2017	Unclear	High	High	Low	Low	Unclear	Unclear

*∗*Risk of bias was assessed with use of the Cochrane risk-of-bias tool.

**Table 3:** Assessment of the quality of cohort studies*∗*.

Study (first author, year)	Selection				Comparability	Outcome			Score
	Representativeness of exposed cohort	Selection of nonexposed cohort	Ascertainment of exposure	Demonstration that outcomes^†^		Assessment	Enough follow-up	Adequacy of follow-up of cohorts	
Kim 2016	☆	-	☆	☆	☆☆	-	☆	☆	7

Lee 2006	☆	-	☆	☆	☆☆	-	☆	-	6

Na 2012	☆	-	☆	☆	☆☆	-	☆	-	6

*∗*The assessment was based on the Newcastle-Ottawa Scale. The full mark of total score is defined as 9; a score of >7 indicates a low risk of bias.

†A demonstration about the outcomes of interest was not present initially.

**Table 4:** Assessment of the quality of case-control studies*∗*.

Study (first author, year)	Selection				Comparability^†^	Exposure			Score
	Definition adequate?	Representativeness of cases	Selection of controls	Definition of controls		Ascertainment of exposure	Same method of ascertainment	Nonresponse rate	
Cho 2013	☆	☆	☆	☆	☆☆	☆	☆	-	8

Tan 2016	☆	☆	-	-	☆☆	☆	-	☆	6

*∗* The assessment was based on the Newcastle-Ottawa Scale. The full mark of total score is defined as 9; a score of >7 indicates a low risk of bias.

† Comparability of cases and controls on the bias of design or analysis.

**Table 5:** Egger's test of publication bias.

Outcome	Std. Eff.	Coef.	Std. Err.	*t*	*P* > |*t*|	[95% Conf. Interval]	
MASI score	Slope	-1.974574	0.5844401	-3.38	0.005	-3.228074	-0.7210746
	Bias	0.6088802	1.955849	0.31	0.760	-3.585999	4.803759
